# Quantitative and functional characteristics of circulating and bone marrow PD-1- and TIM-3-positive T cells in treated multiple myeloma patients

**DOI:** 10.1038/s41598-020-77941-y

**Published:** 2020-11-30

**Authors:** Egor V. Batorov, Tatiana A. Aristova, Vera V. Sergeevicheva, Svetlana A. Sizikova, Galina Y. Ushakova, Natalia V. Pronkina, Irina V. Shishkova, Ekaterina Y. Shevela, Alexander A. Ostanin, Elena R. Chernykh

**Affiliations:** 1 Laboratory of Cellular Immunotherapy, Research Institute of Fundamental and Clinical Immunology, 14 Yadrintsevskaya St, 630099 Novosibirsk, Russian Federation; 2 Department of Hematology and Bone Marrow Transplantation, Research Institute of Fundamental and Clinical Immunology, 14 Yadrintsevskaya St, 630099 Novosibirsk, Russian Federation; 3 Laboratory of Clinical Immunology, Research Institute of Fundamental and Clinical Immunology, 14 Yadrintsevskaya St, 630099 Novosibirsk, Russian Federation

**Keywords:** Myeloma, Tumour immunology

## Abstract

The aim of the present work was to evaluate counts and functional properties of PD-1^+^ and TIM-3^+^ T cells in peripheral blood (PB) and bone marrow (BM) of multiple myeloma (MM) patients following the induction therapy. Sixty patients were enrolled in the study, CD4^+^ and CD8^+^ T cells expressing PD-1 and TIM-3, intracellular production of IFNγ and intracellular expression of Granzyme B were assessed. Relative counts of the majority of circulating PD-1^+^, TIM-3^+^ and PD-1^+^TIM-3^+^ T cells were higher in MM patients with disease progression compared with individuals in remission. Frequencies of almost all evaluated PD-1^+^ and TIM-3^+^ T cell subsets were higher in BM samples compared with PB; circulating CD4^+^PD-1^+^, CD8^+^PD-1^+^, CD8^+^TIM-3^+^, CD8^+^PD-1^+^TIM-3^+^ T cells positively correlated with the same BM subsets. Circulating CD4^+^ T cells, expressing PD-1 and TIM-3 (including co-expressing subset), as well as CD8^+^PD-1^+^TIM-3^+^ T cells, and BM CD8^+^PD-1^+^ T cells correlated with serum B2-M levels. Sufficient frequencies of GrB^+^ and IFNγ^+^ subsets in PD-1-expressing T cells indicated their retained functional properties. TIM-3-expressing T cells and double positive PD-1^+^TIM-3^+^ populations showed diminished cytotoxic and cytokine-producing ability and therefore might be attributed to the exhausted compartment. To identify T cell exhaustion, it is necessary to evaluate T cells co-expressing PD-1, TIM-3 and other inhibitory signal molecules and to study their functional properties. Sustained functionality of PD-1-positive T cells may explain low efficacy and frequent immune-mediated adverse events during anti-PD-1 therapy in MM.

## Introduction

Multiple myeloma (MM) is a malignant plasma cell disease characterized by the uncontrolled proliferation of a B cell precursor clone that differentiates to plasma cells in the bone marrow (BM). A malignant clone in most cases produces a pathological immunoglobulin. The incidence of MM is about 10% of hematologic malignancies^1^. Due to the development and introduction of proteasome inhibitors, immunomodulatory drugs, and high-dose chemotherapy with autologous hematopoietic stem cell transplantation, the median overall survival of MM patients has increased to 5–7 years. Nonetheless, the disease is still considered incurable^1,2^.

Over the last twenty years, a distinct class of inhibitory signal ("checkpoint") molecules (ISMs), such as CTLA-4, PD-1, TIM-3, etc., has been described. ISMs are expressed on T cells and modulate the responses to antigen stimulation under conditions of chronic inflammation in infections and tumor growth. A stable expression of checkpoint molecules on CD4^+^ and CD8^+^ memory T cells is associated with T cell exhaustion. This condition is also characterized by severe decrease in cytotoxic functions and secretion of inflammatory cytokines (IL-2, TNFα, IFNγ)^3^. At the present, a considerable amount of information has been accumulated concerning the blockade of ISMs (especially CTLA-4 and PD-1) by monoclonal antibodies in solid tumors, predominantly of late stages and refractory to other therapies^4^. These studies allowed to understand a critically important role of the checkpoint molecule signaling in the regulation of antitumor immune response.

An increase in T cells expressing ISMs—PD-1, TIM-3, CTLA-4 etc.—was detected in classical Hodgkin lymphoma (HL), several non-Hodgkin lymphomas (NHL) and MM^5–8^. The use of monoclonal antibody drugs blocking ISMs leads to an increase in survival rates in the refractory HL^9,10^. The results of targeted anti-PD-1 therapy for relapsed/refractory NHL and MM remain modest^11,12^. The lack of response may be due to the low expression of PD-L1 by a malignant cell clone and/or tumor microenvironment cells in most types of NHL and MM^11–14^, unlike HL^9,15^. Simultaneously, TIM-3 is considered a perspective target for therapeutic monoclonal antibodies, particularly in combination^16,17^.

However, the expression of one or more ISMs alone is not an indicator of T cell exhaustion. ISMs are expressed by activated T cells with preserved proliferative and cytotoxic functions in normal and pathological conditions^18,19^. Chemotherapy courses can also increase the rate of T cells expressing ISMs^20^.

The aim of the present work was to evaluate quantitative and functional features of PD-1 + and TIM-3 + circulating and BM T cells in MM patients during the induction therapy courses.

## Patients, materials and methods

### Patients and healthy donors

**Figure 1:**
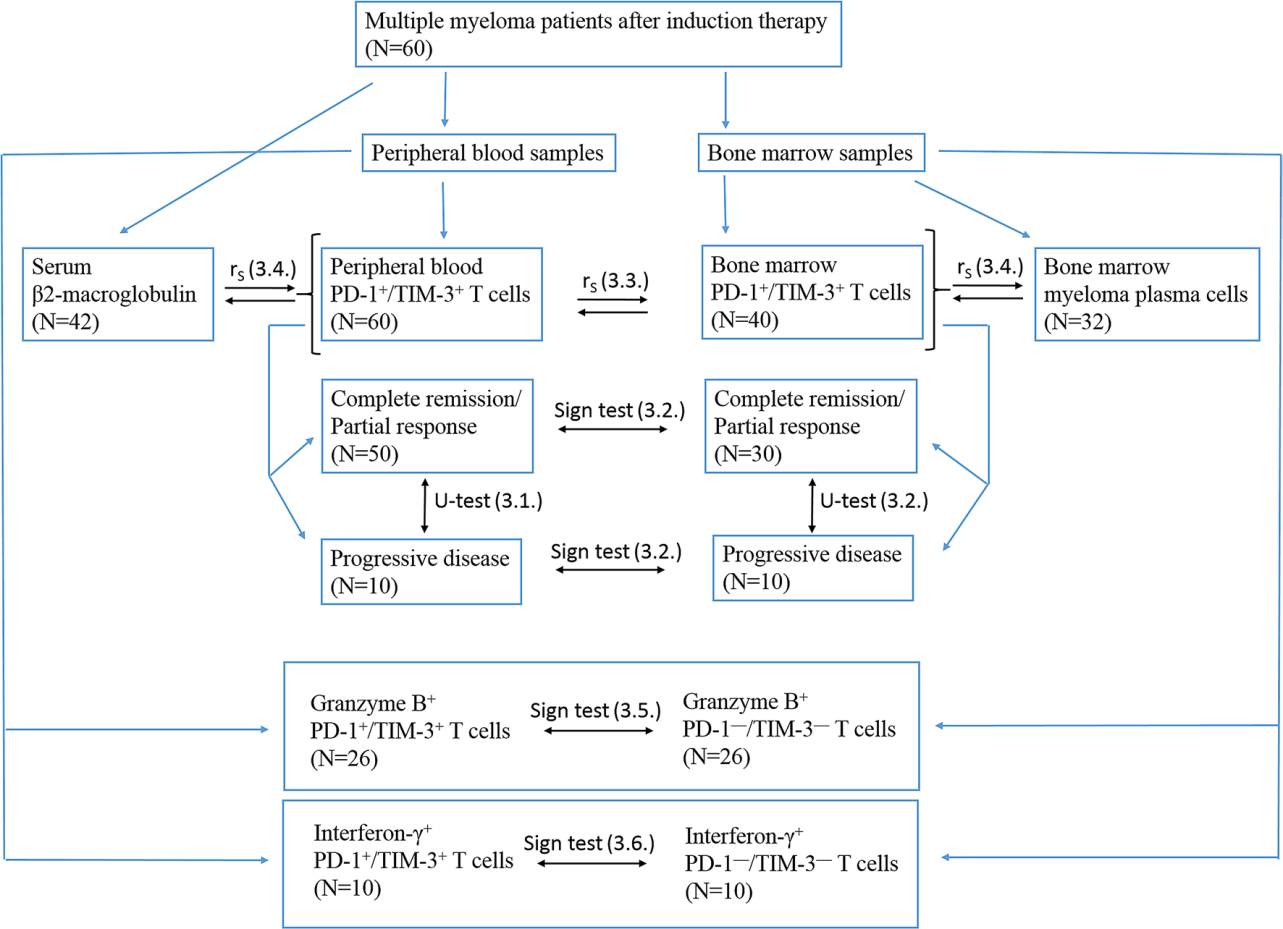
Scheme of the study. Text above black arrows between boxes shows statistical methods used to analyze data; numbers in parentheses above black arrows reflect corresponding subsections in the article. r_S_ indicates Spearman’s rank correlation; sign test, the sign test for paired samples; U-test, the Mann–Whitney U test.

Sixty MM patients who had been treated between April 2018 and September 2019 at the Department of Hematology of Research Institute of Fundamental and Clinical Immunology (Novosibirsk, Russia) and 28 age-matched and sex-matched healthy donors were enrolled in the study. All healthy subjects and patients gave informed consent in accordance with the Declaration of Helsinki of 1975; the local ethics committee approved the study protocol. The patients were staged according to the Durie-Salmon system (1975). Responses were defined according to the International Myeloma Working Group criteria. The exclusion criteria were: any major organ failure, severe drug hypersensitivity, active infectious diseases, major autoimmune diseases. In addition, to avoid any possible effects of activated myeloid cells or intensive lymphocyte expansion, patients receiving granulocyte colony stimulating factor drugs during a month before the analysis and individuals after autologous hematopoietic stem cell transplantation were not included in the study. Baseline characteristics of patients are described in Table 1. The median time from the end of a previous therapy course to analysis was 54 days (interquartile range 24–112 days). Thirteen patients previously received regimens with lenalidomide for 21 days of a 28-day cycle; the median time from the end of the last lenalidomide course to analysis was 52 days (interquartile range: 30—83 days). Patients did not receive lenalidomide maintenance therapy. Treatment regimens are presented in details in Suppl. Table S1. The scheme of the present study is shown in Fig. 1.

**Table 1:** Baseline characteristics of patients.

Characteristic	Value
Age at analysis, years; median (min–max)	51.5 (47—57.5)
Sex, female/male	24/36
**Types**	
IgG	35
IgA	13
Light chain	8
Unknown	4
**Durie-Salmon stage**	
II	18
III	42
**Disease status at analysis**	
Complete remission (including stringent)	11
Partial response, very good partial response	39
Progressive disease	10
**Chemotherapy regimens before analysis**^**1**^	
1	11
2	31
≥ 3	18
Time from the date of diagnosis to analysis, months; median (LQ—UQ)	12.7 (9.2—16.5)

^1^Calculated excluding maintenance therapy with bortezomib and single cyclophosphamide infusion for hematopoietic stem cell mobilization.

### Blood and bone marrow samples

Venous heparinized blood samples obtained from healthy donors were a part of blood donations. Peripheral blood (PB; n = 60) and bone marrow (BM; n = 40) samples from patients were obtained during routine diagnostic procedures, as described earlier^21^. Among others, PB and BM samples of 26 patients were collected with an interval of less than 2 h. Data of patient PB cell counts were obtained from medical records. Peripheral blood and BM samples were processed within an hour of collection. Mononuclear cells (MNCs) were isolated by density gradient centrifugation using Ficoll-Paque (Pharmacia Biotech, Uppsala, Sweden), washed with phosphate buffer solution and with RPMI-1640 Medium supplemented with 0.3 g/L L-glutamine, 25 mM HEPES (Sigma-Aldrich, St. Louis, MO, USA), 100 μg/mL gentamicin (Sigma-Aldrich), 10% autologous serum and underwent immediate analysis by flow cytometry.

### Flow cytometric analysis of T cells

Freshly isolated PB and BM MNCs were stained with the following mouse anti-human monoclonal antibodies according to the manufacturer`s recommendations: CD4-fluorescein isothiocyanate (FITC, clone L200, BD Biosciences, San Jose, CA, USA) or peridinin chlorophyll protein (PerCP, clone OKT4, Biolegend, San Diego, CA, USA); CD8-FITC (clone SK1, BD Biosciences); PD-1-allophycocyanin (APC, clone MIH4, BD Biosciences); TIM-3-phycoerythrin (PE, clone 7D3, BD Biosciences) or PerCP/Cyanine 5.5 (clone F38-2E2, Biolegend); granzyme B (GrB)-PE (clone GB11, BD Biosciences); interferon γ (IFNγ)-PE (cat. 559327, BD Biosciences). VersaLyse Lysing Solution (Beckman Coulter, Marseille, France) was used for erythrocyte lysis according to manufacturer instructions. Before and after incubation with monoclonal antibodies, PB and BM samples were washed with PBS 1500 rpm for 7 min. Samples were stained separately for surface molecules and intracellular markers. As controls were used unstained live cells, “fluorescence-minus-one”, BD CompBeads Anti-mouse Ig, κ/Negative Control Compensation Particles Set (Cat No. 552843) with single colors according to manufacturer instructions. Peripheral blood and BM samples were stained with 7-amino-actinomycin D (7-AAD) immediately prior to flow cytometric analysis to distinguish the dead cells (7-AAD^+^). For intracellular staining, samples were incubated with Transcription Factor Buffer Set (BD Biosciences) according to manufacturer instructions. To determine intracellular expression of IFNγ in T cells, MNCs were stimulated with phorbol 12-myristate 13-acetate (Sigma-Aldrich) (10 ng/mL of cell suspension, 10^6^ cells/mL) and ionomycin (Sigma-Aldrich) (1 μg/mL of cell suspension) for 5 h. After the first hour of incubation, protein transport inhibitors Brefeldin A (BD GolgiPlug, BD Biosciences) and Monensin (BD GolgiStop, BD Biosciences) were added according to the instructions. Samples were analyzed on FACSCalibur and FACSCanto II flow cytometers (BD Biosciences) using CellQuest Pro and FACSDiva software, respectively (BD Biosciences). Lymphocytes were first gated based on forward and side scatter. At least 50,000 gated CD8^+^ T cells per sample were collected for the assay. Gating strategy is presented in Supplementary Fig. S1 online.

### Assessment of serum beta2-microglobulin

Serum samples of treated MM patients were taken after a 12–14 h overnight fasting simultaneously with the PB and BM samples obtained for flow cytometric analysis. Serum beta2-microglobulin (B2-M) was measured using latex particle-enhanced turbidimetry kit (Dako Beta-2-Microglobulin PET Kit, Code No. 0052) on the IMMAGE 800 Immunochemistry System (Beckman Coulter, USA).

### Flow cytometric assessment of bone marrow myeloma plasma cells

Bone marrow myeloma plasma cells (PCs) were determined as described previously^21^. Briefly, fresh BM samples were stained with the following mouse anti-human monoclonal antibodies according to the manufacturer`s recommendations: CD38-FITC or APC, CD56-PE, CD27-peridinin chlorophyll protein complex with cyanin-5.5 (PerCP-Cy5.5), CD138-PerCP-Cy5.5 or APC, CD117- phycoerythrin-cyanin-7 (Pe-Cy7), CD81-allophycocyanin-Hilite 7 (APC-H7), CD19-V450, CD45-V450, cytIgLambda-FITC, cytIgKappa-PE-Cy7 (all MAbs were obtained from BD Biosciences). For intracellular staining, samples were incubated with BD FACS Permeabilizing Solution 2 (BD Biosciences). Samples were analyzed on FACSCanto II flow cytometer using FACSDiva software. For the assessment of minimal residual disease (MRD), at least 10^6^ gated BM cells per sample were acquired. Myeloma plasma cells (PCs) were indicated as CD45^dim^CD38^+^CD138^+^CD56^+^CD19^—^CD117^+^CD27^—^CD81^—^ and were presented as the percentage of all nucleated BM cells. Relative counts of BM myeloma PCs lower than 0.01% were considered MRD-negative. Gating strategy is presented in Supplementary Fig. S2 online.

### Statistical analysis

Statistical analysis was performed using Statistica 6 (StatSoft) package, as described earlier^21^. Data in the text were presented as median and interquartile ranges. The Mann–Whitney U test was used to calculate differences between groups of patients; the sign test was applied to determine differences in parameters within the same (paired) groups of patients. Spearman’s rank correlation was used to evaluate associations for continuous variables. *P* values presented were two-sided. *P* < 0.05 was considered statistically significant. Graphs were prepared using GraphPad Prism 5 software (GraphPad Software, Inc., USA).

### Study approval

The present study was conducted according to the principles of the Declaration of Helsinki. The study protocol was approved by the Research Institute of Fundamental and Clinical Immunology Ethics Committee, and written informed consent was obtained from patients before their inclusion in the study.

## Results

### Frequency of PD-1^+^ and TIM-3^+^ T cells in peripheral blood of multiple myeloma patients

**Figure 2:**
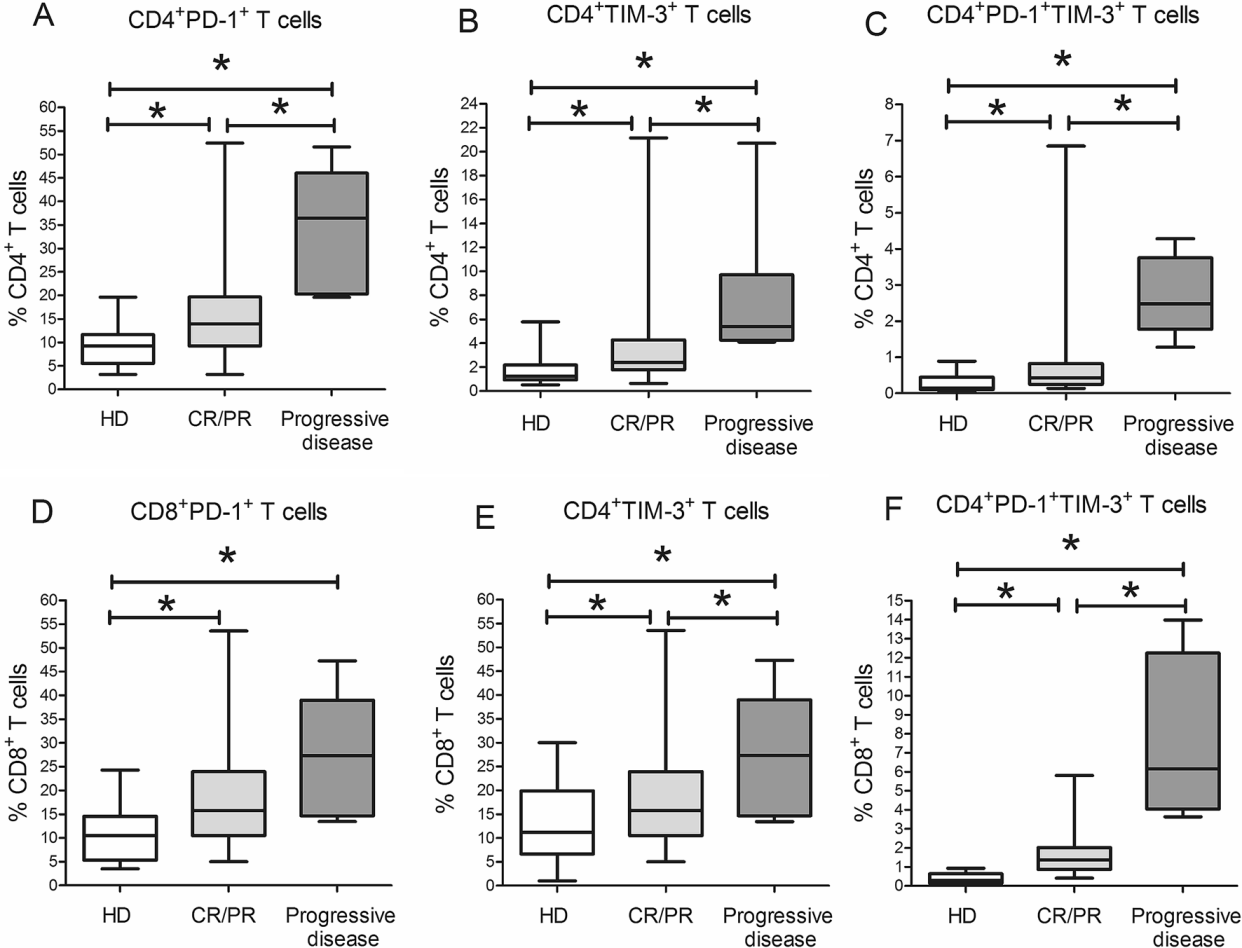
Frequency of circulating PD-1^+^ and TIM-3^+^ T cell subsets in healthy donors and multiple myeloma patients. Graphs show relative counts of peripheral blood CD4^+^PD-1^+^ (**A**), CD4^+^TIM-3^+^ (**B**), CD8^+^PD-1^+^ (**D**), CD8^+^TIM-3^+^ (**E**) T cells and double positive CD4^+^PD-1^+^TIM-3^+^ (**C**) and CD8^+^PD-1^+^TIM-3^+^ (**F**) T cell subsets in healthy donors (HD, *n* = 28, white boxes), MM patients in complete remission or partial response (CR/PR,* n* = 50, light grey boxes) and MM patients with progressive disease (*n* = 10, dark grey boxes). Data are expressed as median, interquartile range and range of minimum and maximum values. *P* values are assessed with Mann–Whitney U-test (*P_*U*_ < 0.05). Generated using GraphPad Prism 5.0 (GraphPad Software, Inc., San Diego, CA, USA; https://www.graphpad.com/).

We first analyzed relative and absolute counts of circulating CD4^+^ and CD8^+^ T cells expressing PD-1 or TIM-3 in MM patients, divided into remission (complete remission (CR) and partial response (PR)) and progression groups. Relative counts of PD-1^+^ and TIM-3^+^ cells in circulating CD8^+^ and CD4^+^ T cells, as well as the percentages of circulating CD4^+^PD-1^+^, CD4^+^TIM-3^+^, CD8 + PD-1^+^ and CD8^+^TIM-3^+^ T cell subsets among total lymphocytes, in both groups of patients were significantly higher compared with healthy donor values (Fig. 2, Table 2). Simultaneously, due to the higher absolute lymphocyte counts in healthy donors, the absolute values of only CD4^+^TIM-3^+^ T cell subset remained statistically higher in patients compared with donors (Suppl. Table S2).

**Table 2:** Counts of circulating PD-1^+^ and TIM-3^+^ T cell subsets in multiple myeloma patients.

Cell subset	Healthy donors (*n* = 28)	Patients in CR/PR (*n* = 50)	Patients with progressive disease (*n* = 10)
CD4^+^PD-1^+^, %	4.4 (2.6–5.3)	6.1 (4.7–9.8)*	11.8 (7.3–13.4)*^#^
CD4^+^TIM-3^+^, %	0.6 (0.4–1.0)	1.3 (0.8–2.3)*	2.0 (1.2–2.5)*
CD8^+^PD-1^+^, %	1.6 (1.1–3.2)	2.9 (1.6–4.1)*	3.9 (1.3–12.2)*
CD8^+^TIM-3^+^, %	3.1 (1.8–5.4)	3.8 (2.4–5.7)*	8.9 (4.8–12.0)*^#^

Relative counts are presented as the percentages of lymphocytes.

Data are presented as median (interquartile range). *P* values are assessed with Mann–Whitney U-test.

*CR* complete remission; *PR* partial response.

*P_*U*_ < 0.05 between healthy donors and patients.

^#^*P*_*U*_ < 0.05 between patients in CR/PR and patients with progressive disease.

PD-1^+^ and TIM-3^+^ subsets of CD4^+^ T cells and TIM-3^+^ subset of CD8^+^ T cells were higher in patients with progressive disease compared with the individuals in remission (Fig. 2). These differences were also demonstrated for the relative counts of circulating CD4^+^PD-1^+^ and CD8^+^TIM-3^+^ T cell subsets among total lymphocytes (Table 2). There were no difference in absolute counts of PD-1^+^ and TIM-3^+^ T cells between the patients in remission and the ones with progressive MM, probably because of the lower absolute lymphocyte counts in the last group (Suppl. Table S2).

Relative counts of double positive (PD-1^+^TIM-3^+^) subsets in circulating CD4^+^ and CD8^+^ T cells were substantially lower comparing with single positive cell populations (PD-1 or TIM-3). Peripheral blood of patients with progressive disease contained significantly higher proportions of PD-1^+^TIM-3^+^ subsets compared with the remission group (Fig. 2). In healthy donors, co-expression of PD-1 and TIM-3 on circulating T cells was detected at a very low level (Fig. 2, Supplementary Fig. S3 online).

### Relative counts of PD-1^+^ and TIM-3^+^ T cells in the BM of MM patients in different disease phases

**Figure 3:**
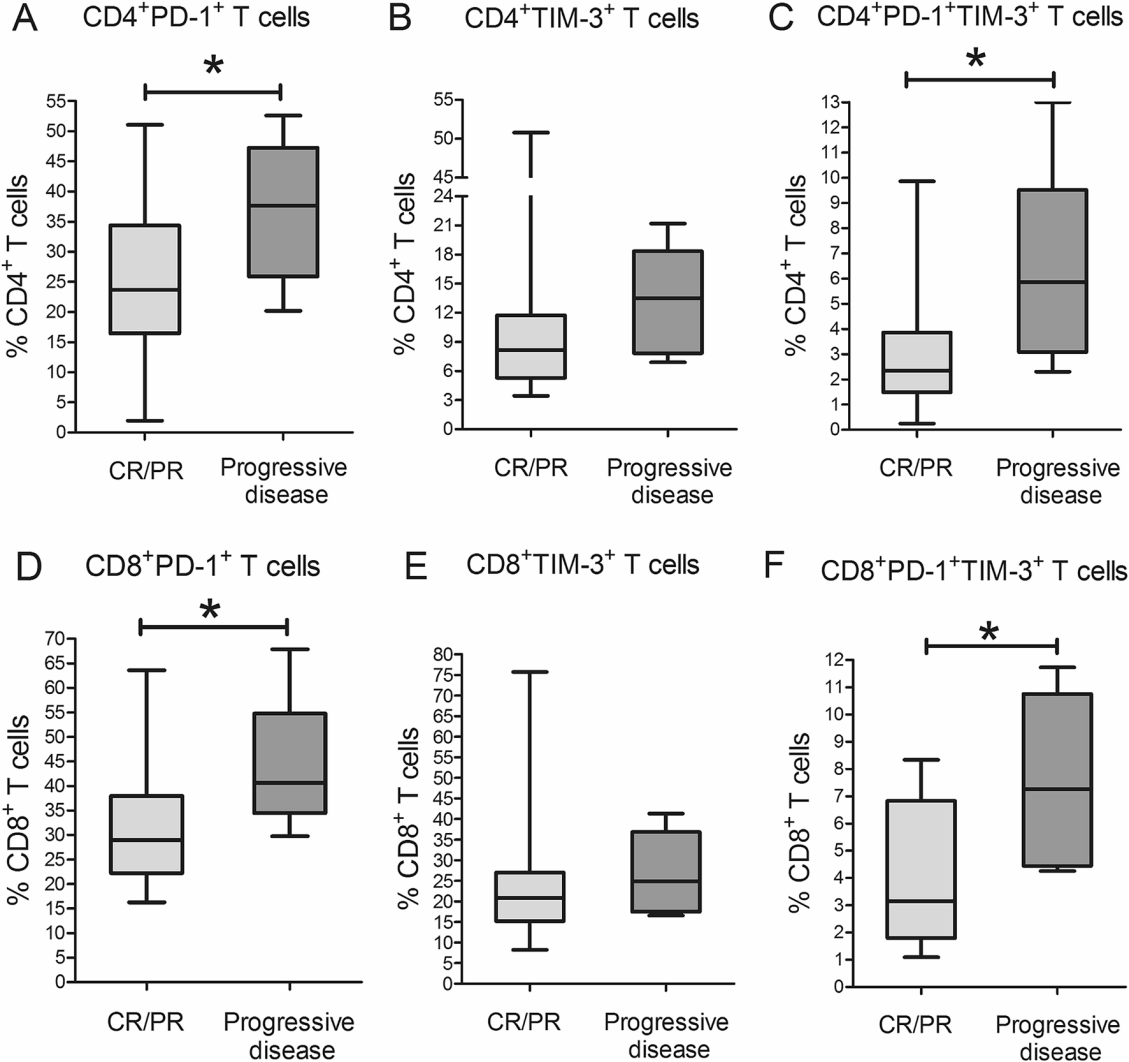
Frequency of PD-1^+^ and TIM-3^+^ T cell subsets in bone marrow samples of healthy donors and multiple myeloma patients. Graphs show relative counts bone marrow CD4^+^PD-1^+^ (**A**), CD4^+^TIM-3^+^ (**B**), CD8^+^PD-1^+^ (**D**), CD8^+^TIM-3^+^ (**E**) T cells and double positive CD4^+^PD-1^+^TIM-3^+^ (**C**) and CD8^+^PD-1^+^TIM-3^+^ (**F**) T cell subsets in MM patients in complete remission or partial response (CR/PR,* n* = 30, light grey boxes) and MM patients with progressive disease (*n* = 10, dark grey boxes). Data are expressed as median, interquartile range and range of minimum and maximum values. *P* values are assessed with Mann–Whitney U-test (*P_*U*_ < 0.05). Generated using GraphPad Prism 5.0 (GraphPad Software, Inc., San Diego, CA, USA; https://www.graphpad.com/).

The main site of tumor growth in MM is the bone marrow, therefore we evaluated the frequency of PD-1^+^ or TIM-3^+^ subsets of CD4^+^ and CD8^+^ T cells in BM samples obtained from MM patients. PD-1^+^ subsets of both CD4^+^ and CD8^+^ T cells were higher in the patients with progressive disease compared with the individuals in remission (Fig. 3). On the contrary, only relative count of CD8^+^TIM-3^+^ T cells within BM lymphocytes was higher in patients with progressive disease (Table 3).

**Table 3:** Counts of bone marrow PD-1^+^ and TIM-3^+^ T cell subsets in multiple myeloma patients.

Cell subset	Patients in CR/PR (*n* = 30)	Patients with progressive disease (*n* = 10)	*P*_U_
CD4^+^PD-1^+^, %	5.3 (3.7–9.0)	7.1 (2.7–8.7)	0.75
CD4^+^TIM-3^+^, %	2.2 (1.2–3.2)	1.8 (1.6–3.0)	0.79
CD8^+^PD-1^+^, %	4.9 (3.3–7.1)	6.4 (6.2–11.4)	0.076
CD8^+^TIM-3^+^, %	3.5 (2.5–4.4)	5.7 (4.7–6.5)	0.033
CD4^+^PD-1^+^TIM-3^+^, %	0.5 (0.4–0.8)	1.5 (1.1–2.5)	0.0052
CD8^+^PD-1^+^TIM-3^+^, %	0.7 (0.3–0.9)	1.3 (0.9–1.6)	0.089

Relative counts are presented as the percentages of bone marrow lymphocytes.

Data are presented as median (interquartile range). *P* values are assessed with Mann–Whitney U-test.

*CR* complete remission; *PR* partial response.

TIM-3^+^ subset of CD4^+^ T cells and PD-1^+^ subset of CD8^+^ T cells were higher in the BM compared with PB in both remission and progression groups (Supplementary Fig. S4). PD-1^+^ subset of CD4^+^ T cells obtained from BM samples were higher compared with its counterpart in PB of patients in CR/PR (Supplementary Fig. S4). There were no statistical differences in the frequency of TIM3^+^ subset in CD8^+^ T cells between PB and BM in both groups of patients (Supplementary Fig. S4).

Relative counts of double positive PD-1^+^TIM-3^+^ subsets in both CD4^+^ and CD8^+^ T cells were higher in BM samples of the patients with progressive disease comparing with the remission group (Fig. 3). The frequency of BM CD4^+^PD-1^+^TIM-3^+^ T cells among BM lymphocyte pool was also higher in the individuals with progressive disease; for BM CD8^+^PD-1^+^TIM-3^+^ T cell subset the same difference appeared as a trend (Table 3). BM samples contained considerably higher counts of PD-1^+^TIM-3^+^ subsets in CD4^+^ and CD8^+^ T cells compared with the circulating counterparts in patients in CR/PR (Supplementary Fig. S4). In progression group, PD-1^+^TIM-3^+^ subset in CD4^+^ T cells was significantly higher in BM samples compared with PB, while CD8^+^PD-1^+^TIM-3^+^ T cells was equally high both in BM and in PB (Supplementary Fig. S4).

### Correlation between frequencies of PB and BM PD-1^+^ and TIM-3^+^ T cells

We measured the percentage of PD-1^+^ and TIM-3^+^ T cells in PB and BM collected simultaneously (with an interval of less than 2 h) in 26 MM patients. There were significant positive correlations between the majority (except CD4^+^TIM-3^+^ and CD4^+^PD-1^+^TIM-3^+^) of circulating PD-1^+^ and TIM-3^+^ subsets and the residual BM counterparts both in T cell populations and in whole lymphocyte pool (Table 4). Therefore, we suggest that BM PD-1^+^ and TIM-3^+^ T cells might be potential sources for the appropriate circulating subsets.

**Table 4:** Correlation analysis between circulating and bone marrow PD-1^+^ and TIM-3^+^ T cell subsets in multiple myeloma patients.

Cell subset in PB and BM	% of CD4^+^ or CD8^+^ T cells	% of lymphocytes
CD4^+^PD-1^+^	r_S_ = 0.43, *P* = 0.027, *n* = 26	r_S_ = 0.50, *P* = 0.0090, *n* = 26
CD4^+^TIM-3^+^	r_S_ = 0.020, *P* = 0.92, *n* = 26	r_S_ = 0.38, *P* = 0.056, *n* = 26
CD8^+^PD-1^+^	r_S_ = 0.51, *P* = 0.0078, *n* = 26	r_S_ = 0.52, *P* = 0.0066, *n* = 26
CD8^+^TIM-3^+^	r_S_ = 0.52, *P* = 0.0062, *n* = 26	r_S_ = 0.61, *P* = 0.00091, *n* = 26
CD4^+^PD-1^+^TIM-3^+^	r_S_ = 0.25, *P* = 0.24, *n* = 26	r_S_ = 0.34, *P* = 0.10, *n* = 26
CD8^+^PD-1^+^TIM-3^+^	r_S_ = 0.46, *P* = 0.025, *n* = 26	r_S_ = 0.46, *P* = 0.026, *n* = 26

Spearman’s rank correlation was used to evaluate associations for the studied variables.

### Correlations between markers of tumor burden and frequencies of PB and BM PD-1^+^ and TIM-3^+^ T cells

Further, we tried to evaluate a possible association between tumor load and relative counts of PD-1^+^ and TIM-3^+^ T cells. Serum B2-M level and CD45^dim^CD38^+^CD138^+^CD56^+^CD19^−^CD117^+^CD27^−^CD81^−^ BM myeloma PCs were selected as markers of tumor burden and were assessed in 48 and 38 treated MM patients, respectively. T cell populations and malignant PCs were analyzed in the same BM sample; only samples of MRD-positive patients were assessed.

Patients with MM progression had higher serum B2-M levels compared with CR/PR group (10.5 mg/L (7.2–19.1 mg/L; *n* = 6 vs 2.3 mg/L (1.9–2.7 mg/L); *n* = 42, *P*_U_ < 0.00001). Similarly, this group was characterized with higher relative counts of BM malignant PCs (5.2% of nucleated BM cells (2.3–20.4%); *n* = 6 vs 0.3% (0.1–1.3%); *n* = 32, *P*_U_ = 0.0053). In addition, serum B2-M levels positively correlated with BM malignant PCs: r_S_ = 0.50, *P* = 0.0014 (*n* = 38).

We found, that serum B2-M levels correlated with relative counts of circulating PD-1^+^ and TIM-3^+^ subsets of CD4^+^ T cells (r_S_ = 0.31, *P* = 0.034, and r_S_ = 0.29, *P* = 0.050, respectively, *n* = 48) and double positive PD-1^+^TIM-3^+^ populations of both CD4^+^ and CD8^+^ T cells (r_S_ = 0.35, *P* = 0.021, and r_S_ = 0.46, *P* = 0.0019, respectively, *n* = 48). Between studied BM lymphocytes only relative count of PD-1^+^ subset of CD8^+^ T cells positively correlated with serum B2-M levels: r_S_ = 0.48.

*P* = 0.0037, *n* = 48. We did not show significant associations between the percentage of BM myeloma PCs and the assessed T cell subsets.

### Cytotoxic potential of circulating and BM PD-1^+^ and TIM-3^+^ T cells

To evaluate functional activity of ISM expressing T cells, we firstly tested cytotoxic ability of PB and BM PD-1^+^ and TIM-3^+^ CD8^+^ T cells by intracellular production of GrB.

**Figure 4:**
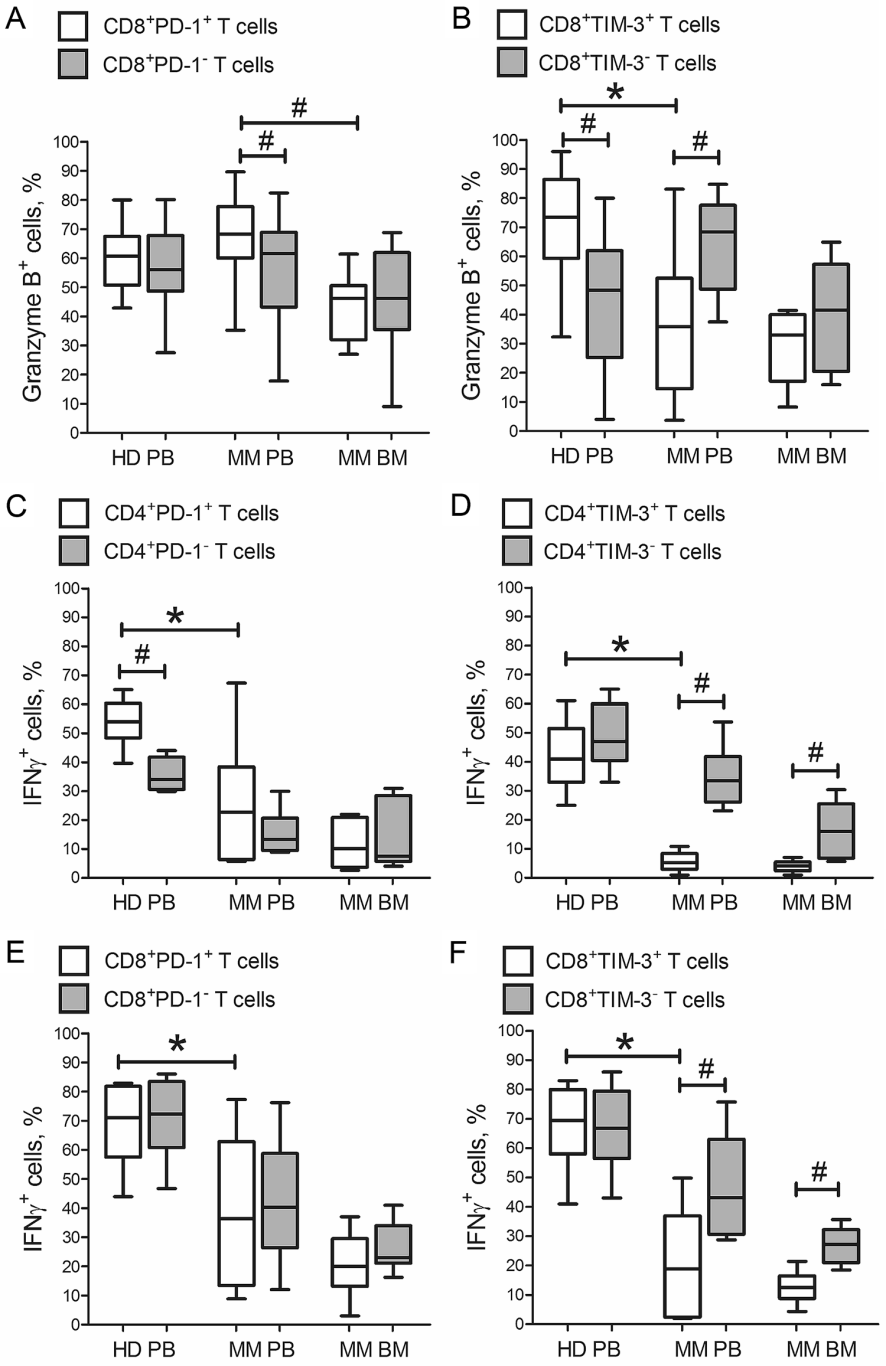
Frequency of granzyme B^+^ and IFNγ^+^ cells in PD-1- and TIM-3-positive and negative CD8^+^ T cell subsets in healthy donors and multiple myeloma patients. Graphs A-B show relative counts of granzyme B^+^ cells in (**A**) PD-1- and (**B**) TIM-3-positive (white boxes) and negative (grey boxes) CD8^+^ T cells in PB of healthy donors (HD PB, *n* = 15) and in PB and BM of MM patients (MM PB and MM BM, respectively,* n* = 26). Graphs **C**–**F** show relative counts of IFNγ^+^ cells in (**C**,**E**) PD-1- and (D, F) TIM-3-positive (white boxes) and negative (grey boxes) CD4^+^ (**C**,**D**) and CD8^+^ (**E**,**F**) T cells in PB of healthy donors (HD PB, *n* = 10) and in PB and BM of MM patients (MM PB and MM BM, respectively,* n* = 10). Data are expressed as median, interquartile range and range of minimum and maximum values. *P* values are assessed with Mann–Whitney U-test (*P_*U*_ < 0.05) between independent groups and sign test (^#^*P* < 0.05) between paired groups. Generated using GraphPad Prism 5.0 (GraphPad Software, Inc., San Diego, CA, USA; https://www.graphpad.com/).

In healthy donors, the frequency of GrB^+^ cells did not differ between circulating CD8^+^PD-1^+^ and CD8^+^PD-1^−^ T cells (Fig. 4A). Meanwhile, relative count of GrB^+^ cells was significantly higher in CD8^+^TIM-3^+^ T cells compared with TIM-3-negative subset (Fig. 4B). The percentage of GrB^+^ cells in CD8^+^PD-1^+^TIM-3^+^ subset was just slightly lower: 52.1% (44.3–62.8%), *n* = 15.

The frequency of GrB^+^ cells in PB CD8^+^PD-1^+^ T cells of MM patients was comparable to the donor values and significantly higher compared with CD8^+^PD-1^−^ T cells (Fig. 4A, Supplementary Fig. S5A). On the contrary, relative count of GrB^+^ cells in PB CD8^+^TIM-3^+^ T cells of MM patients was significantly lower compared with the same subset of healthy donors and patient CD8^+^TIM-3^−^ T cell subset (Fig. 4B, Supplementary Fig. S5C). The proportion of GrB^+^ cells in CD8^+^PD-1^+^TIM-3^+^ T cell subset was also decreased: 28.7% (24.4–48.3%), *n* = 26. There were no differences in T cell cytotoxic potential between CR/PR patients (n = 22) versus progressing individuals (n = 4).

The percentage of GrB^+^ cells was significantly lower in BM CD8^+^PD-1^+^ T cells of MM patients, compared with the same subset in PB (Fig. 4A). The proportion of GrB^+^ cells in BM CD8^+^PD-1^+^ and CD8^+^PD-1^-^ T cells did not significantly differ (Fig. 4A). Relative count of GrB^+^ cells in CD8^+^TIM-3^+^ T cells from BM of MM patients was as low as in PB. The percentage of GrB^+^ cells in BM CD8^+^TIM-3^+^ and CD8^+^TIM-3^-^ T cells was nearly equal (Fig. 4B), and GrB^+^ cells in double positive CD8^+^PD-1^+^TIM-3^+^ subset was slightly decreased: 27.3% (15.0–60.9%), *n* = 26.

Thereby, in healthy individuals, the expression of PD-1 and TIM-3 by CD8^+^ T cells, including the majority of double positive cells, associated with preserved (or even enhanced) cytotoxic potential. In MM patients, CD8^+^PD-1^+^ T cells also retained high proportion of GrB^+^ T cells, while CD8^+^TIM-3^+^ and especially CD8^+^PD-1^+^TIM-3^+^ T cells possessed diminished cytotoxic ability.

### Interferon γ-producing ability of circulating and BM PD-1^+^ and TIM-3^+^ T cells

Further, we focused on the assessment of intracellular IFNγ by PB and BM PD-1^+^ and TIM-3^+^ T cells. In healthy donors, the frequency of IFNγ^+^ cells was relatively high in ISM-expressing T cells, especially in CD8^+^ T cells. Relative count of IFNγ^+^ cells was higher in CD4^+^PD-1^+^ T cells compared with PD-1-negative subset (Fig. 4C). The frequency of IFNγ^+^ cells did not differ between following T cell subsets: CD4^+^TIM-3^+^ and CD4^+^TIM-3^−^ T cells, CD8^+^PD-1^+^ and CD8^+^PD-1^−^ T cells, CD8^+^TIM-3^+^ and CD8^+^TIM-3^−^ T cells (Fig. 4D–F).The percentage of IFNγ^+^ cells in CD8^+^PD-1^+^TIM-3^+^ subset was also high: 70.7% (54.0–78.9%), *n* = 10. IFNγ^+^ cells in CD4^+^PD-1^+^TIM-3^+^ cells did not differ from CD4^+^ single-positive subsets: 37.1% (28.8–49.0%), *n* = 10.

In MM patients, the frequencies of IFNγ^+^ cells in circulating CD4^+^PD-1^+^ and CD8^+^PD-1^+^ T cells were significantly lower compared with the same donor cell subsets but remained comparable to CD4^+^PD-1^−^ and CD8^+^PD-1^−^ T cells, respectively (Fig. 4C,E, Supplementary Fig. S5B). On the contrary, relative counts of IFNγ^+^ cells in PB CD4^+^TIM-3^+^ and CD8^+^TIM-3^+^ T cells of MM patients were dramatically diminished compared with both the donor values and patient TIM-3^−^ T cell subsets (Fig. 4D,F, Supplementary Fig. S5D). The proportions of IFNγ^+^ cells in double positive PD-1^+^TIM-3^+^ CD4^+^ and CD8^+^ T cells were also decreased: 7.0% (4.2–9.8%), *n* = 10, and 11.5% (9.6–37.5%), *n* = 10, respectively.

The percentage of IFNγ^+^ cells did not significantly differ between BM PD-1^+^ or TIM-3^+^ T cells and the same subsets in PB of MM patients (Fig. 4C–F). The proportion of IFNγ^+^ cells in BM PD-1^+^ CD4^+^ and CD8^+^ T cells did not significantly differ from PD-1-negative populations (Fig. 4C,E). Relative counts of IFNγ^+^ cells in CD4^+^TIM-3^+^ and CD8^+^TIM-3^+^ T cells from BM of MM patients were significantly lower compared with CD4^+^TIM-3^-^ and CD8^+^TIM-3^-^ subsets, respectively (Fig. 4D,F). The percentages of IFNγ^+^ cells in BM double positive CD4^+^PD-1^+^TIM-3^+^ and CD8^+^PD-1^+^TIM-3^+^ subsets were low: 4.5% (2.2–5.9%), *n* = 10, and 10.0% (7.4–11.2%), *n* = 10.

## Discussion

The increased expression of ISM ligands (for example, PD-L1 and PD-L2 for PD-1) by malignant cells and/or tumor microenvironment is an effective way of escaping from immune surveillance. Therapeutic monoclonal antibodies (anti-PD-1, anti-CTLA-4) competitively block ligand-receptor interactions, preventing the attenuation of antitumor immune response. Attempts to treat MM (including newly diagnosed and smoldering MM) with monoclonal anti-PD-1 antibodies did not show relevant clinical results while immuno-mediated adverse reactions were frequent^11,12^. However, studies in this direction are ongoing. The present work was devoted to a characterization of quantitative and functional properties of circulating and BM-derived T cells expressing PD-1 and TIM-3 in MM patients receiving induction therapy courses.

We did not evaluate the expression of PD-1 and TIM-3 receptors by T cells of patients at the time of diagnosis, since previous studies have repeatedly demonstrated an increase in T cells expressing PD-1 and other ISMs in newly diagnosed MM patients comparing with healthy volonteers^5,7,8,22^. Further, according to Görgün et al. and Zelle-Rieser et al., PD-1^+^ T cell counts of chemo-naïve patients were as high as their counterparts in treated individuals with refractory MM^5,8^. The data obtained by Paiva et al. stand partially apart; according to the authors, T cells expressing PD-1 did not differ between healthy controls, newly diagnosed and MRD-negative patients, and were increased in individuals with progressive MM and even MRD-positive disease^13^.

Firstly, we described in details the frequencies of PD-1^+^, TIM-3^+^ and double-positive PD-1^+^TIM-3^+^ T cells in CD4^+^ and CD8^+^ populations as well as their proportions in total lymphocytes. Relative counts of studying subsets were higher in MM patients compared with healthy donors. Concerning PB CD4^+^PD-1^+^ and especially CD8^+^PD-1^+^ T cells, our findings are in agreement with the literature data^5,8^. Circulating TIM-3-positive and double-positive PD-1^+^TIM-3^+^ T cells had been described much poorer in MM patients. Tan and al. reported that TIM-3^+^CD57^+^ subsets of CD3^+^ and CD8^+^ T cells as well as PD-1^+^TIM-3^+^CD3^+^ T cells were significantly increased in PB from 10 newly diagnosed patients with MM compared with healthy individuals^7^. Furthermore, we showed for the first time the increase of circulating PD-1^+^ and TIM-3^+^ T cells (except PD-1^+^ subset of CD8^+^ T cells) in patients with progressive MM compared with ones in CR/PR.

In the majority of MM cases, tumor growth and associated cellular interactions proceed within BM. We found the significant increase of PD-1^+^ and double-positive PD-1^+^TIM-3^+^ subsets in BM CD4^+^ and CD8^+^ T cells in the patients with progressive disease compared with the individuals in remission. Paiva et al.^13^ and Chang et al.^23^ also had shown higher PD-1^+^ T cell counts in MM patients at relapse compared with patients in remission (according to Paiva et al.—only for MRD-negative cases). In our study, counts of TIM-3^+^ subsets in BM T cells did not differ depending on MM status, but, contrariwise, the percentage of CD8^+^TIM-3^+^ T cells among BM lymphocytes was higher in patients with progressive disease, presumably, due to the higher CD8^+^ cell proportion in the BM of that group.

Frequencies of almost all evaluated PD-1^+^ and TIM-3^+^ T cell subsets were higher in BM samples compared with PB, except CD8^+^TIM-3^+^ T cells. Also, in progression group, proportions of PD-1^+^ subset in CD4^+^ T cells and PD-1^+^TIM-3^+^ subset of CD8^+^ T cells were high in PB and therefore did not differ from BM specimens. Higher level of T cells expressing ISMs in the BM of MM patients seems logical, as BM is the site of tumor, stromal and immune cell interactions. Nonetheless, previously published data are contradictory. Zelle-Rieser et al. did not find differences of PD-1^+^ populations in CD4^+^ and CD8^+^ T cells between BM aspirates and PB of MM patients (*n* = 7–12)^8^. Tan et al. showed higher CD8^+^PD-1^+^ subsets in BM samples compared with PB of untreated MM patients (*n* = 10), while CD4^+^PD-1^+^ cells and TIM-3^+^ populations (including double-positive PD-1^+^TIM-3^+^ T cells) were nearly equal for both of these sources^7^.

In our study, circulating CD4^+^PD-1^+^, CD8^+^PD-1^+^, CD8^+^TIM-3^+^, CD8^+^PD-1^+^TIM-3^+^ T cells correlated with the same BM subsets. Therefore, PB T cells, expressing ISMs in MM might partially originate from affected BM and, supposedly, reflect the severity of T cell dysfunction. It is difficult to determine, whether PB PD-1^+^ and TIM-3^+^ T cells play any sufficient role in MM pathogenesis, or these cells are only “washed away” from BM.

Further, we investigated associations between tumor load and relative counts of circulating and BM PD-1^+^ and TIM-3^+^ T cells. Serum B2-M level and the frequency of BM myeloma PCs were selected as markers of tumor burden^24–28^. In our study, circulating CD4^+^ T cells, expressing PD-1 and TIM-3 (including co-expressing cells), as well as CD8^+^PD-1^+^TIM-3^+^ T cells, and BM CD8^+^PD-1^+^ T cells correlated with serum B2-M levels. Previously, Chang et al. also showed that BM CD3^+^PD-1^+^ T cells in BM of both untreated and relapsed/refractory IgG type MM patients correlated with their serum B2-M concentrations and myeloma cell counts^23^. On the contrary, Sponaas et al. could not find a correlation between the percent of BM PD1^+^ CD8^+^ T cells and the number of BM PCs in heterogeneous group of MM patients; increased number of BM PD1^+^CD8^+^EOMES^high^Tbet^low^ cells was described only in individuals with tumor load above 10% PCs^29^. To our slight surprise, we also did not indicate associations between counts of PD-1- and TIM-3-positive T cells and myeloma PCs, although BM T cells and malignant PCs had been evaluated from the same samples. It can be presumed that the circulating ISM-expressing T cells (as well as serum B2-M) represent a rough and indirect but systemic reflection of whole body tumor load. Simultaneously, residual malignant PC and PD-1^+^/TIM-3^+^ T cell interactions are complex and most likely not always linear, even though both cell compartments increase with disease progression.

The functional impairment is a hallmark of T cell exhaustion. To evaluate cytotoxic and cytokine-producing potential of circulating and BM PD-1^+^ and TIM-3^+^ T cells, we tested intracellular GrB and IFNγ production. The ability to produce IFNγ is discontinued last after other cytokines^30^. We found a pronounced discrepancy in functional abilities of T cells, expressing the studying molecules. A substantial proportion of both circulating and BM CD8^+^PD-1^+^ T cells of MM patients were GrB-positive. Relative counts of GrB^+^ cells in PB CD8^+^PD-1^+^ T cells were comparable to the donor values and even higher than in the paired CD8^+^PD-1^-^ subset. Frequencies of circulating IFNγ^+^ cells in both CD4^+^PD-1^+^ and CD8^+^PD-1^+^ T cells of MM patients were lower compared with the same donor subsets, but did not differ from patient PD-1-negative T cells. On the contrary, relative counts of GrB^+^ cells in CD8^+^TIM-3^+^ T cells and IFNγ^+^ cells in CD4^+^TIM-3^+^ and CD8^+^TIM-3^+^ T cells were diminished compared with the same subsets of healthy donors and paired TIM-3^-^ T cells. The percentages of GrB^+^ and IFNγ^+^ subsets in double positive PD-1^+^TIM-3^+^ T cells were nearly equal to the same in TIM-3^+^ T cells. Bone marrow PD-1^+^ and TIM-3^+^ T cells of MM patients entirely repeated the described functional properties of circulating populations.

Preserved functional activity of PD-1 expressing T cells or at least their several subsets was reported for MM^29^, acute myeloid leukemia^18^ and several non-hematological malignancies^31–35^. Relatively early publications had attributed PD-1^+^ T cells with preserved functional potential to the activated population^18^. Recent studies, based on single cell RNA sequencing technologies and multicolor flow cytometry, resolved to accurately define “dysfunctional” (”exhausted”) tumor infiltrating PD-1^+^ T cells at different states of functional impairment, where “early dysfunctional” (or “progenitor exhausted”) cells expressed intermediate levels of PD-1 and showed maintained or even increased cytotoxic, IFNγ-producing and proliferative capacity^32–35^.

In contrast, the reduced functional activity of CD8^+^TIM-3^+^ and CD4^+^TIM-3^+^ T cells allowed to assume that the expression of TIM-3 molecule is more associated with T cell exhaustion than PD-1. Previously, Lee et al. described significantly decreased proliferative capacity, production of cytokines, GrB and perforin by CD8^+^TIM-3^+^PD-1^−^ and double positive CD8^+^TIM-3^+^PD-1^+^ T cells compared to CD8^+^TIM-3^−^PD-1^+^ cells in patients with malignant Schwannomas^31^. Similar data for hematological diseases have not been published, and the regulatory role of TIM-3 in MM requires further investigations.

Simultaneously, low counts of GrB^+^ and IFNγ^+^ cells in double positive TIM-3^+^PD-1^+^ T cells are in agreement with literature data as co-expression of two or more ISMs is a marker of T cell exhaustion^3,36^. The mentioned above recent transcriptomic and cytometric studies confirmed the predominant co-expression of TIM-3 and high levels of PD-1 by “late dysfunctional”/”terminal exhausted” T cells with dramatically diminished effector functions in melanoma^32,33^, non-small cell lung cancer^34^, hepatocellular carcinoma^35^.

To summarize the above, our data demonstrated that relative counts of the majority of circulating PD-1^+^, TIM-3^+^ and double-positive PD-1^+^TIM-3^+^ T cells were higher in MM patients with disease progression compared with individuals in remission. Frequencies of almost all evaluated PD-1^+^ and TIM-3^+^ T cell subsets were higher in BM samples compared with PB; circulating CD4^+^PD-1^+^, CD8^+^PD-1^+^, CD8^+^TIM-3^+^, CD8^+^PD-1^+^TIM-3^+^ T cells correlated with the same BM subsets. In addition, circulating CD4^+^ T cells, expressing PD-1 and TIM-3 (including double positive subset), as well as CD8^+^PD-1^+^TIM-3^+^ T cells, and BM CD8^+^PD-1^+^ T cells correlated with serum B2-M levels. Sufficient frequencies of GrB^+^ and IFNγ^+^ subsets in PD-1-expressing T cells indicated their retained functional properties, while TIM-3-expressing T cells and double positive PD-1^+^TIM-3^+^ populations showed diminished cytotoxic and cytokine-producing ability and therefore might be attributed to the exhausted compartment. Schematic representation of summarized findings is presented in Supplementary Fig. S6 online. To identify the state of T cell exhaustion, it is necessary to evaluate T cells co-expressing PD-1, TIM-3 and other ISMs and/or to study their functional properties. Retained functional activity of PD-1-positive T cells may explain low efficacy and frequent immune-mediated adverse events during anti-PD-1 therapy in multiple myeloma.

## Supplementary information

Supplementary Information.

## Data Availability

The authors declare that the main data supporting the results of the present study are available within the article and its Supplementary Information files. Some data analysed during the current study contain personal medical information and are available from the corresponding author on reasonable request.

## Abbreviations

B2-M: Beta2-microglobulin
BM: Bone marrow
CR: Complete remission
GrB: Granzyme B
HDC: High-dose chemotherapy
HL: Hodgkin lymphoma
IFNγ: Interferon γ
ISM: Inhibitory signal molecule
MM: Multiple myeloma
MNC: Mononuclear cell
MRD: Minimal residual disease
NHL: Non-Hodgkin lymphoma
PB: Peripheral blood
PC: Plasma cell
PR: Partial response
