# Supplementary material for: Quantitative and functional characteristics of circulating and bone marrow PD-1- and TIM-3-positive T cells in treated multiple myeloma patients
Source: Sci Rep. 2020 Nov 30;10:20846. doi: 10.1038/s41598-020-77941-y (PMC7704628; doi:10.1038/s41598-020-77941-y)
Supplement: Supplementary file 1 — Supplementary Information. [file 41598_2020_77941_MOESM1_ESM.pdf]

Quantitative and functional characteristics of circulating and bone marrow PD-1- and TIM-3-positive T cells in treated multiple myeloma patients

Egor V. Batorov[1], Tatiana A. Aristova[2], Vera V. Sergeevicheva[2], Svetlana A. Sizikova[2], Galina Y. Ushakova[2], Natalia V. Pronkina[3], Irina V. Shishkova[3], Ekaterina Y. Shevela[1], Alexander A. Ostanin[1], Elena R. Chernykh[1]

[1]Laboratory of Cellular Immunotherapy, Research Institute of Fundamental and Clinical Immunology, 630099, 14 Yadrintsevskaya St, Novosibirsk, Russian Federation

[2]Department of Hematology and Bone Marrow Transplantation, Research Institute of Fundamental and Clinical Immunology, 630099, 14 Yadrintsevskaya St, Novosibirsk, Russian Federation

[3]Laboratory of Clinical Immunology, Research Institute of Fundamental and Clinical Immunology, 630099, 14 Yadrintsevskaya St, Novosibirsk, Russian Federation

Corresponding author: Egor Batorov

Research Institute of Fundamental and Clinical Immunology, 630099, 14 Yadrintsevskaya St, Novosibirsk, Russian Federation. Phone No.: +7 (383) 228-21-01; Fax No.: +7(383) 222-70-28. E-mail: Ebatorov@gmail.com

Supplementary table S1. Prior regimens at baseline.

<table>
<tr><td></td><td>VCD</td><td>PAD</td></tr>
<tr><td>Patients received only one chemo regimen, N</td><td>5</td><td>6</td></tr>
<tr><td rowspan="2"></td><td colspan="2" rowspan="2">VCD + PAD</td><td colspan="2">VCD/PAD</td></tr>
<tr><td>+ (V)RD</td><td>+ Kd</td></tr>
<tr><td>Patients received 2 regimens, N</td><td colspan="2">28</td><td>2</td><td>1</td></tr>
<tr><td rowspan="2"></td><td colspan="2" rowspan="2">VCD ± PAD ± BBD + RD</td><td colspan="3">VCD ± PAD ± BBD</td></tr>
<tr><td>+Kd</td><td>+ DCEP</td><td>+ other</td></tr>
<tr><td>Patients received ≥ 3 regimens, N</td><td colspan="2">10</td><td>1</td><td>3</td><td>5</td></tr>
<tr><td colspan="6">BBD indicates bendamustine, bortezomib and dexamethasone; DCEP, dexamethasone, cyclophosphamide, etoposide, cisplatin; Kd, carfilzomib and dexamethasone; PAD, bortezomib (PS-341), doxorubicin and dexamethasone; RD, lenalidomide and dexamethasone; VCD, bortezomib, cyclophosphamide and dexamethasone.</td></tr>
</table>

Supplementary table S2. Absolute counts of circulating PD-1^{+} and TIM-3^{+} T cell subsets in multiple myeloma patients

Cell subset	Healthy donors (n = 28)	Patients in CR/PR (n = 50)	Patients with progressive disease (n = 10)
Lymphocytes, × 10^{9}/L	1.63 (1.47—1.92)	1.32 (1.07—1.95)*	0.61 (0.53—1.20)*$^{\#}$
$CD4^{+}PD\text{-}1^{+}$, /μL	67 (39—102)	82 (60—117)	81 (39—138)
$CD4^{+}TIM\text{-}3^{+}$, /μL	10 (7—12)	19 (12—31)*	16 (12—30)*
$CD8^{+}PD\text{-}1^{+}$, /μL	32 (20—50)	36 (22—57)	35 (10—146)
$CD8^{+}TIM\text{-}3^{+}$, /μL	50 (33—64)	57 (29—77)	72 (35—147)

Data are presented as median (interquartile range). *P* values are assessed with Mann–Whitney U-test.

*$P_U < 0.05$ between healthy donors and patients.

$^{\#}P_U < 0.05$ between patients in CR/PR and patients with progressive disease.

CR, complete remission; PR, partial response.

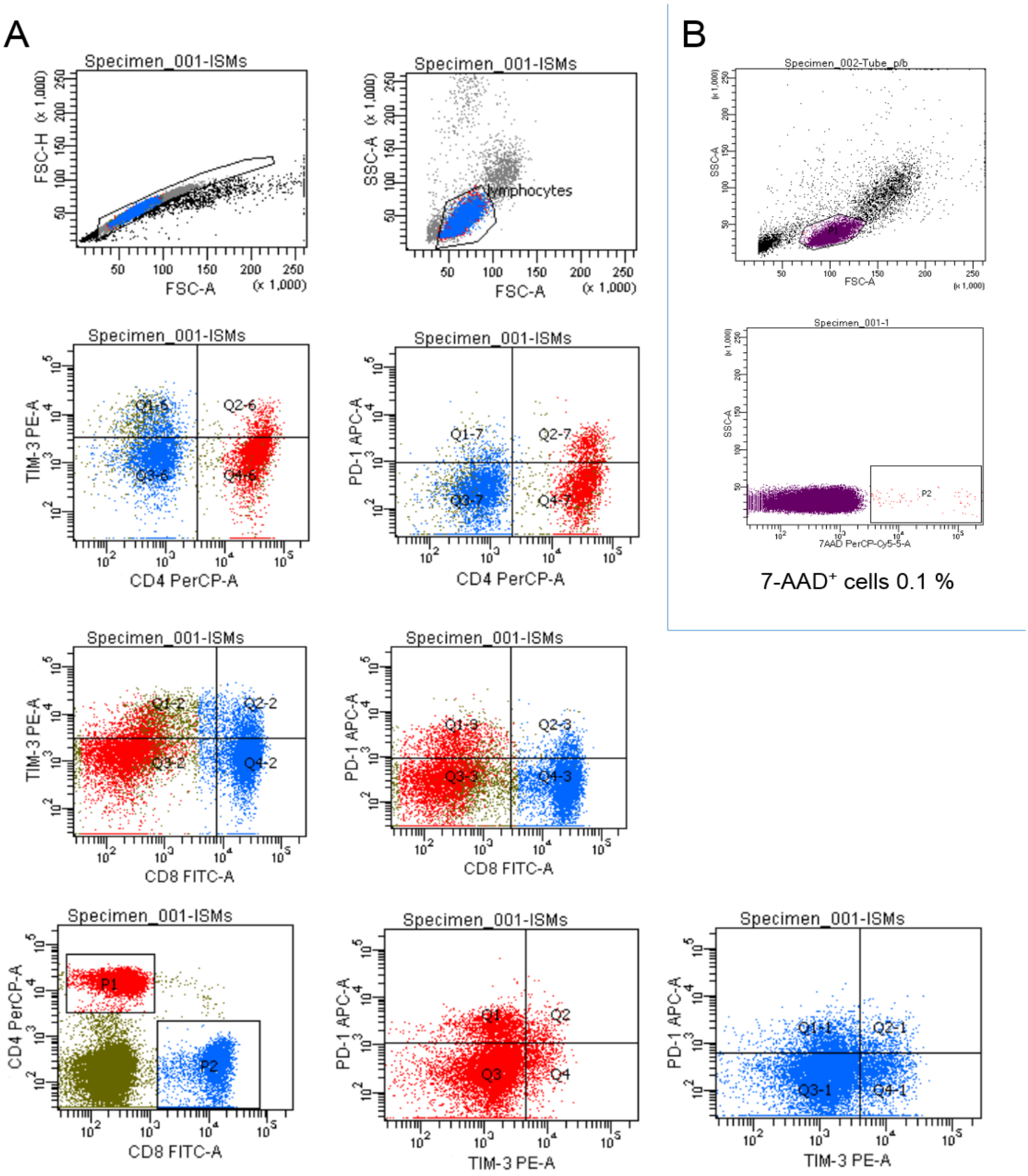

Supplementary Figure S1. Gating strategy and flow cytometric characteristics of PD-1$^+$ and TIM-3$^+$ T cell subsets. Cell doublets were excluded from the analysis based on characteristics of forward scatter area (FSC-A) and height (FSC-H); lymphocyte gate was selected (A, upper row). 7-AAD staining was performed separately to assess the frequencies of dead cells (7-AAD$^+$) (B). Relative counts of CD4$^+$ and CD8$^+$ (A, the 2nd and 3rd rows, respectively) T cells expressing TIM-3 and PD-1 (upper right quadrants) were studied in lymphocyte region. Double positive PD-1$^+$TIM-3$^+$ subsets (A, bottom row, the 2nd and 3rd graphs, upper right quadrants) were measured in CD4$^+$ and CD8$^+$ T cell regions (A, P1 and P2 gates, marked in red and blue, respectively). Data of representative multiple myeloma patient are presented. Created with FACSDiva software (BD Biosciences, San Jose, CA, USA; https://www.bdbiosciences.com/).

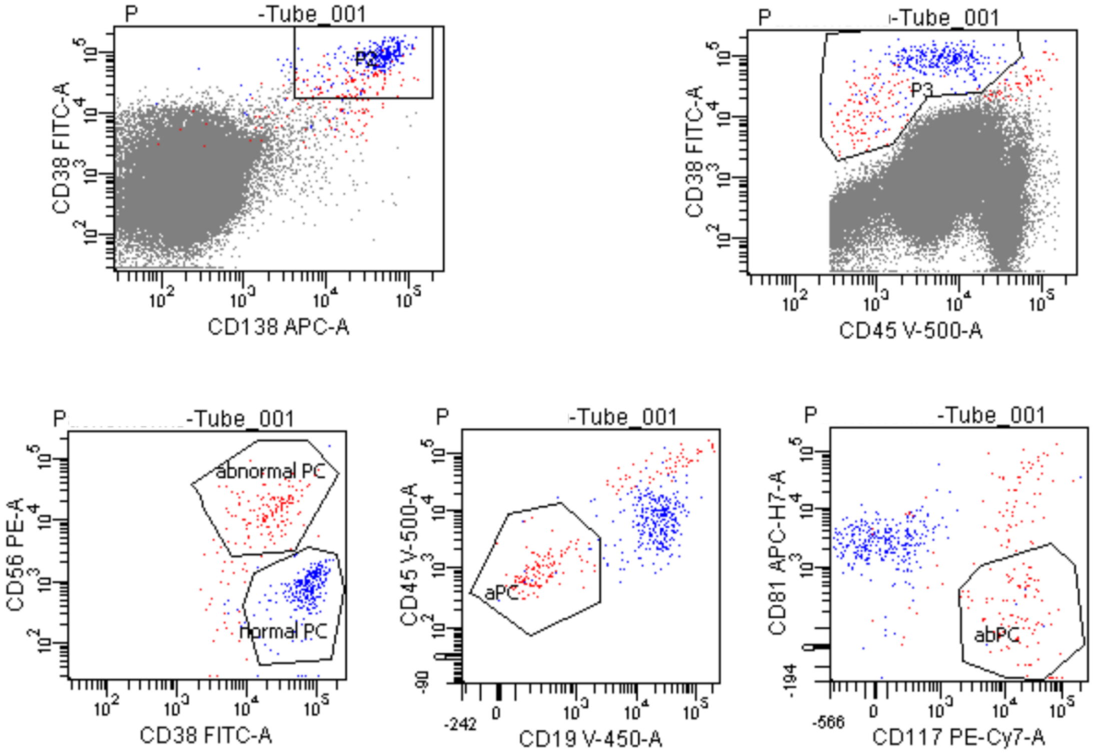

	FITC	PE	PerCP-Cy5.5	PE-Cy7	APC	APC-H7	V450	V510
**Tube 1**	***CD38***	***CD56***	***CD27***	***CD117***	***CD138***	***CD81***	***CD19***	***CD45***
**Tube 2**	***cyt IgL***	***CD56***	***CD138***	***cyt IgK***	***CD38***		***CD19***	***CD45***

Supplementary figure S2. Gating strategy of bone marrow myeloma plasma cells.

Created with FACSDiva software (BD Biosciences, San Jose, CA, USA; https://www.bdbiosciences.com/).

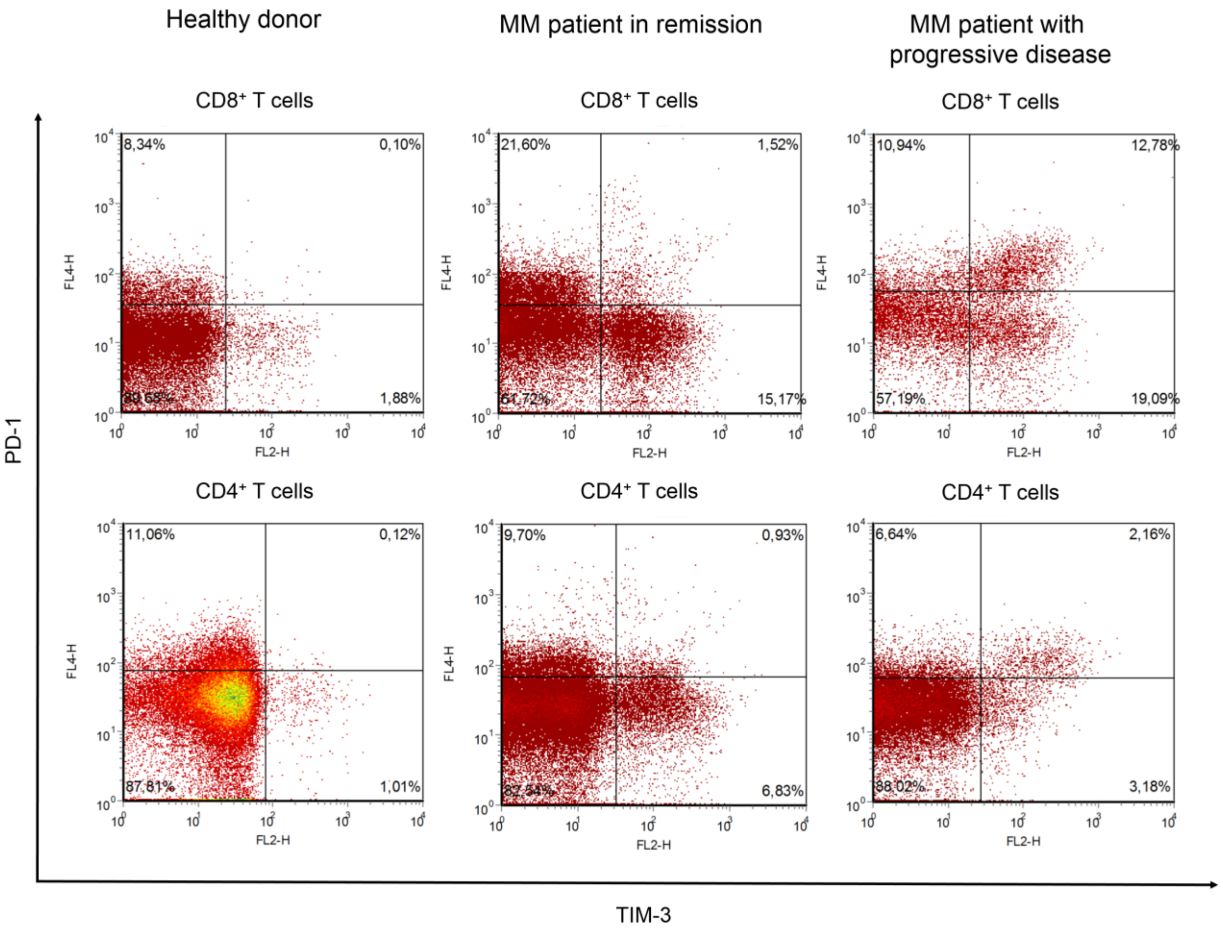

Supplementary Figure S3. Flow cytometry characteristic of CD8$^+$PD-1$^+$TIM-3$^+$ and CD4$^+$PD-1$^+$TIM-3$^+$ T cells in a healthy donor and multiple myeloma patients. Relative counts of double positive PD-1$^+$TIM-3$^+$ subsets (upper right quadrants) among circulating CD8$^+$ and CD4$^+$ T cells are shown. Flow cytometric data of representative healthy donor, MM patient in remission and MM patient with progressive disease are presented. Created with FCS Express V3 (De Novo Software, LA, CA, USA; https://denovosoftware.com/).

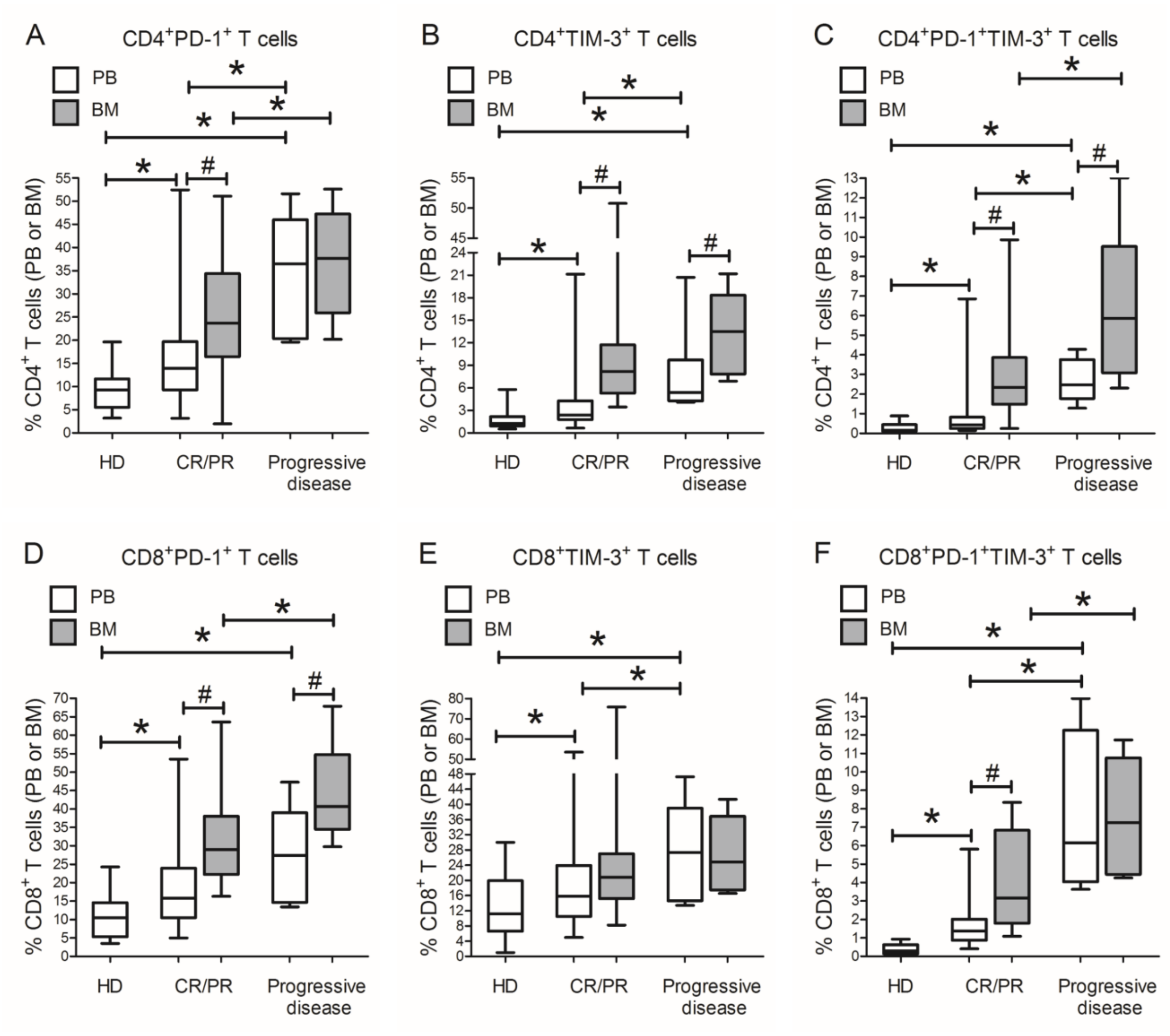

Supplementary Figure S4. Frequency of PD-1$^+$ and TIM-3$^+$ T cell subsets in healthy donors and multiple myeloma patients. Graphs show relative counts of peripheral blood (white boxes) and bone marrow (grey boxes, for MM patients only) CD4$^+$PD-1$^+$ (A), CD4$^+$TIM-3$^+$ (B), CD8$^+$PD-1$^+$ (D), CD8$^+$TIM-3$^+$ (E) T cells and double positive CD4$^+$PD-1$^+$TIM-3$^+$ (C) and CD8$^+$PD-1$^+$TIM-3$^+$ (F) T cell subsets in healthy donors (HD, n = 28), MM patients in complete remission or partial response (CR/PR, n = 50) and MM patients with progressive disease (n = 10).

Data are expressed as median, interquartile range and range of minimum and maximum values. P values are assessed with Mann–Whitney U-test ($*P_U < 0.05$) between independent groups and sign test ($^{\#}P < 0.05$) between paired groups. Generated using GraphPad Prism 5.0 (GraphPad Software, Inc., San Diego, CA, USA; https://www.graphpad.com/).

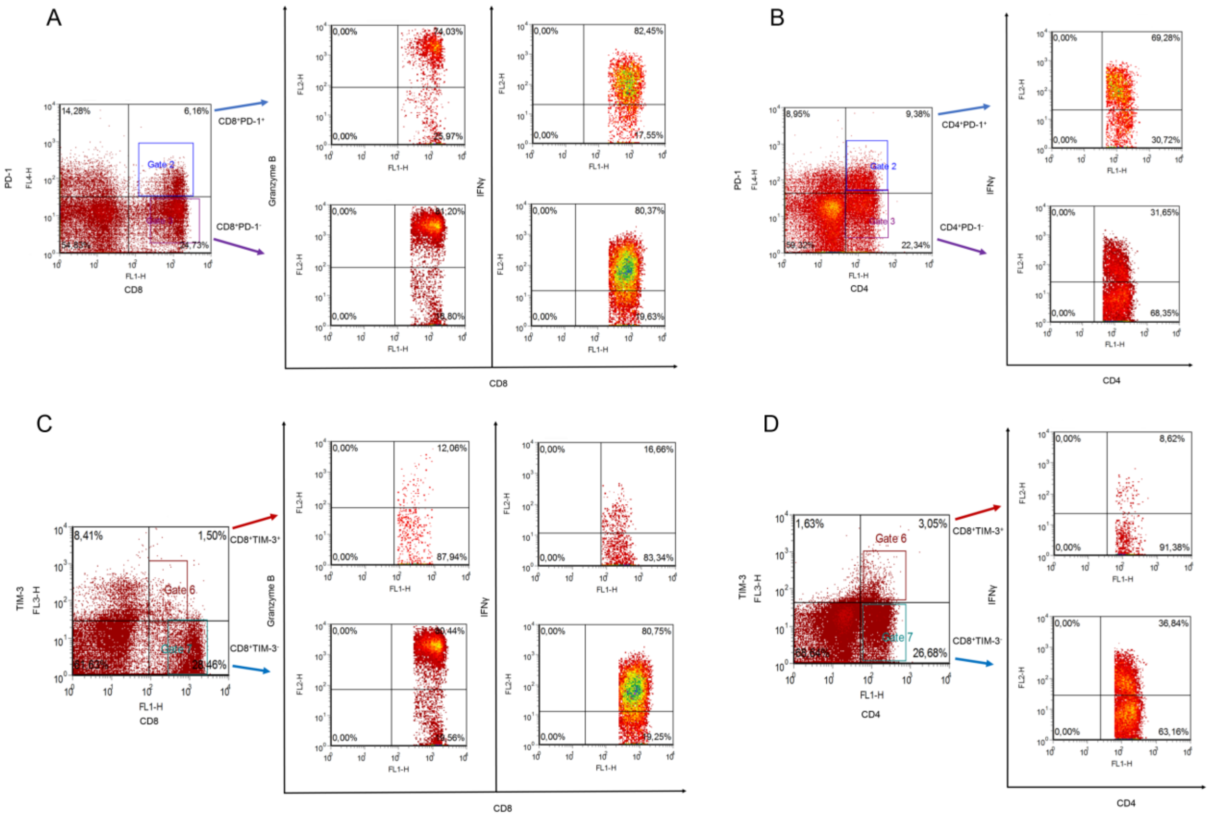

Supplementary figure S5. Flow cytometric characteristics of the intracellular content of granzyme B and interferon-γ in circulating T cells, depending on the surface expression of inhibitory signaling molecules PD-1 and TIM-3.

Relative counts of granzyme B-positive and interferon-γ-positive cells among $CD8^{+}PD\text{-}1^{+}$ and $CD4^{+}PD\text{-}1^{+}$ T cells did not differ from PD-1-negative subsets (A, B). Among $CD8^{+}TIM3^{+}$ and $CD4^{+}TIM\text{-}3^{+}$ T cells, the proportion of granzyme B-positive and interferon-γ-positive cells was lower compared with TIM-3-negative subsets (C, D).

Data of representative multiple myeloma patient are presented. Created with FCS Express V3 (De Novo Software, LA, CA, USA; https://denovosoftware.com/).

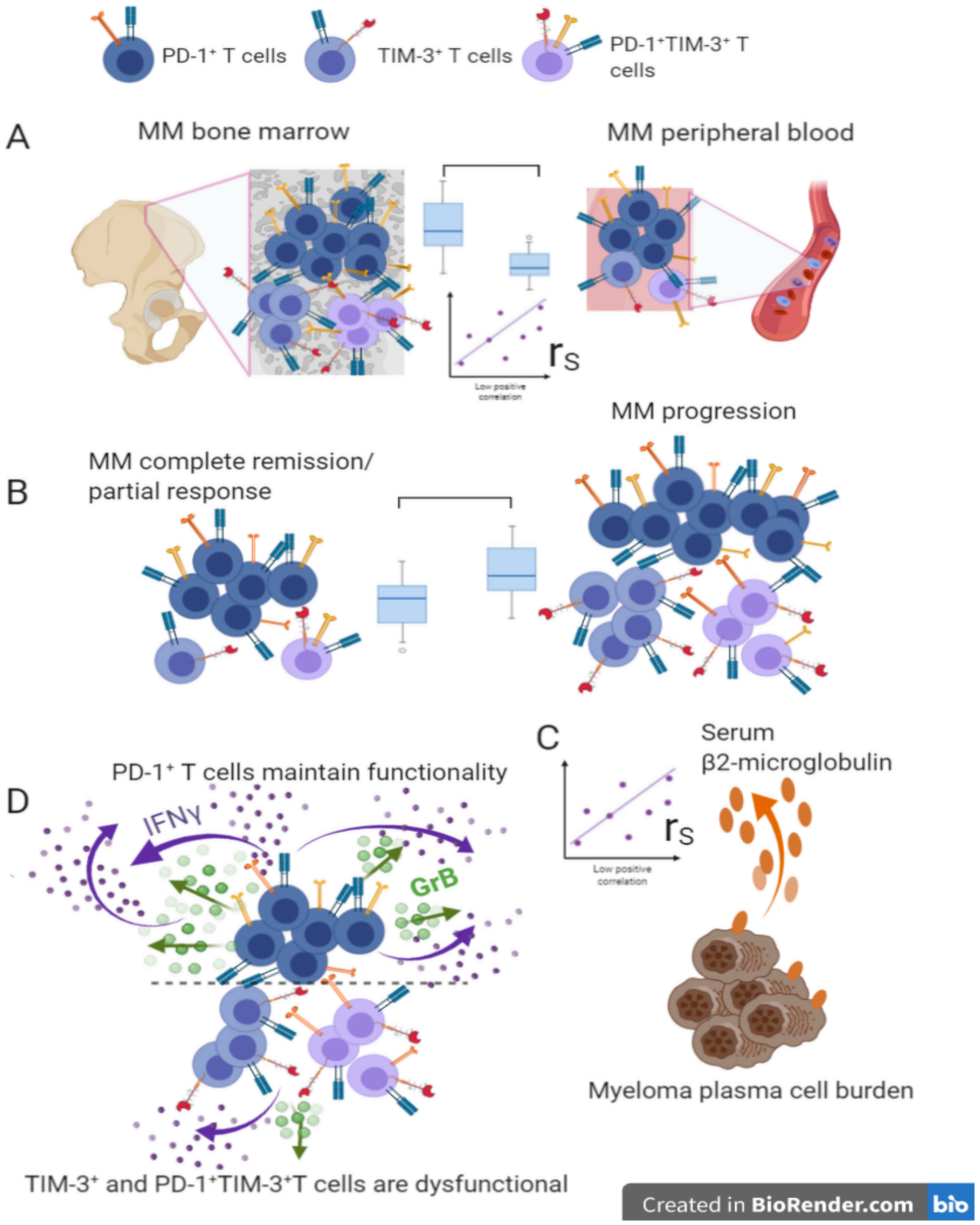

Supplementary Figure S6. Simplified representation of PD-1$^+$ and TIM-3$^+$ T cell compartments in peripheral blood and bone marrow of treated multiple myeloma patients.

According to our findings, frequencies of almost all evaluated PD-1$^+$ and TIM-3$^+$ T cell subsets were higher in bone marrow (BM) samples compared with peripheral blood (PB); circulating CD4$^+$PD-1$^+$, CD8$^+$PD-1$^+$, CD8$^+$TIM-3$^+$, CD8$^+$PD-1$^+$TIM-3$^+$ T cells positively correlated with the same BM subsets (A). The majority of PD-1$^+$ and/or TIM-3$^+$ and double positive PD-1$^+$TIM-3$^+$ T cell subsets were higher in MM patients with disease progression compared with individuals in remission (B). Serum beta2-microglobulin level (a marker of tumor burden) correlated with relative counts of circulating PD-1$^+$ and TIM-3$^+$ subsets of CD4$^+$ T cells and double positive PD-1$^+$TIM-3$^+$ populations of both CD4$^+$ and CD8$^+$ T cells (C). Sufficient proportion of PD-1$^+$ T cells produces GrB and IFNγ, while TIM-3$^+$ T cells and double positive PD-1$^+$TIM-3$^+$ populations show diminished cytotoxic and cytokine-producing potential (D). Created with BioRender.com
